# Safety climate, safety climate strength, and length of stay in the NICU

**DOI:** 10.1186/s12913-019-4592-1

**Published:** 2019-10-22

**Authors:** Daniel S. Tawfik, Eric J. Thomas, Timothy J. Vogus, Jessica B. Liu, Paul J. Sharek, Courtney C. Nisbet, Henry C. Lee, J. Bryan Sexton, Jochen Profit

**Affiliations:** 1 Division of Pediatric Critical Care Medicine, Department of Pediatrics, Stanford University School of Medicine, 770 Welch Road, Suite 435, Stanford, CA 94304 USA; 2 The McGovern Medical School at The University of Texas Health Science Center at Houston, Houston, TX USA; 3 The University of Texas – Memorial Hermann Center for Healthcare Quality and Safety, Houston, TX USA; 4 Graduate School of Management, Vanderbilt University, Nashville, TN USA; 5 Perinatal Epidemiology and Health Outcomes Research Unit, Division of Neonatology, Department of Pediatrics, Stanford University School of Medicine, Stanford, CA USA; 6 California Perinatal Quality Care Collaborative, Stanford, CA USA; 7 Center for Quality and Clinical Effectiveness, Lucile Packard Children’s Hospital, Palo Alto, CA USA; 8 Division of Pediatric Hospitalist Medicine, Department of Pediatrics, Stanford University, Stanford, CA USA; 9 Department of Psychiatry, Duke University Health System, Duke University School of Medicine, Durham, NC USA; 10 Duke Center for Healthcare Safety and Quality, Duke University Health System, Durham, NC USA

**Keywords:** Safety climate, Safety climate strength, Length of stay, Quality of care, Neonatology

## Abstract

**Background:**

Safety climate is an important marker of patient safety attitudes within health care units, but the significance of intra-unit variation of safety climate perceptions (safety climate strength) is poorly understood. This study sought to examine the standard safety climate measure (percent positive response (PPR)) and safety climate strength in relation to length of stay (LOS) of very low birth weight (VLBW) infants within California neonatal intensive care units (NICUs).

**Methods:**

Observational study of safety climate from 2073 health care providers in 44 NICUs. Consistent perceptions among a NICU’s respondents, i.e., safety climate strength, was determined via intra-unit standard deviation of safety climate scores. The relation between safety climate PPR, safety climate strength, and LOS among VLBW (< 1500 g) infants was evaluated using log-linear regression. Secondary outcomes were infections, chronic lung disease, and mortality.

**Results:**

NICUs had safety climate PPRs of 66 ± 12%, intra-unit standard deviations 11 (strongest) to 23 (weakest), and median LOS 60 days. NICUs with stronger climates had LOS 4 days shorter than those with weaker climates. In interaction modeling, NICUs with weak climates and low PPR had the longest LOS, NICUs with strong climates and low PPR had the shortest LOS, and NICUs with high PPR (both strong and weak) had intermediate LOS. Stronger climates were associated with lower odds of infections, but not with other secondary outcomes.

**Conclusions:**

Safety climate strength is independently associated with LOS and moderates the association between PPR and LOS among VLBW infants. Strength and PPR together provided better prediction than PPR alone, capturing variance in outcomes missed by PPR. Evaluations of NICU safety climate consider both positivity (PPR) and consistency of responses (strength) across individuals.

## Background

Error reduction in health care relies on a shared understanding that the organization prioritizes safety behaviors and practices above competing interests, and that it recognizes, rewards, and supports such behaviors by all employees. (1, 2) In other words, it relies on a well-developed safety climate. In order to achieve this, two separate but related elements are required. First, the actions of organizational leaders must emphasize safety in an obvious way. Second, the workforce must largely agree in its assessment of the emphasis on safety. Workforce assessments of patient safety climate are typically measured through safety climate surveys, which have been used extensively in health care research. (3–15)

Ample research suggests that a more positive safety climate is associated with a host of workforce and patient outcomes. Correspondingly, safety climate is increasingly being understood as a driver of quality of care, (2, 6, 12, 14, 16–19) and benchmarking of safety climate is now a focus in the United States by the Joint Commission, (20) the Agency for Healthcare Research and Quality, (21) and Leapfrog. (22) However, some recent work also suggests (albeit with low response rates) that safety climate may not be a key mechanism underpinning safety and is otherwise difficult to shape. (23) Failures to modify safety climate may be a function of how it is assessed and interpreted. That is, prior research has focused on the level of safety climate (i.e., how positive it is) and largely assumed consistency.

However, safety climate perceptions may range from highly consistent to highly variable within an organization, with the degree of consistency termed safety climate strength. (2, 24) Climate strength derives from foundational psychological work by Walter Mischel which finds that situations differ in their ambiguity and “situational strength,” with strong situations creating clarity that leads individuals with shared experiences to perceive events similarly and have uniform expectations regarding the most appropriate behaviors. (25) In contrast, weak situations are highly ambiguous, individuals have different perceptions, and inconsistent or non-existent behavioral expectations arise. Thus, when perceptions of the level of safety climate are consistent (i.e., strong safety climate), the priority placed on safety and the attendant behavioral expectations are clear, and more uniform behavior is likely to result.^22,41^ Additionally, climate strength also increases over time with strong climates persisting, while weaker ones may not.^41^ Therefore, the relationship between safety climate and outcomes is expected to be enhanced by a stronger safety climate. The more consistent the workforce safety experience is, the more likely it is to behave consistently as a collective with regard to safety. (26)

Since the Institute of Medicine report highlighting medical errors in 1999, (27) substantial efforts have focused on the reduction of medical errors in the United States. In parallel, there has also been a focus, particularly in the NICU, on the costs of preterm birth, with mean in-hospital costs of $76,000 to $159,000 (in 2018 dollars) per very low birth weight (VLBW) infant. (28) Length of stay (LOS) carries particular importance as a quality measure for VLBW infants, as it serves multiple roles: a marker of value for health policy makers and payers, a competitive benchmark for payment, a measure of quality and family centered care, an indicator of safety lapses, and a target for quality improvement. (29) Consequently, there is great interest by hospitals operating NICUs and health policy makers alike to deliver safer care in ways that also reduce LOS.

Although safety climate levels vary among NICUs, (10, 30, 31) safety climate strength profiles of NICUs are unexamined, and the relation between safety climate strength and neonatal quality of care is unknown. Thus, the objective of this study was to analyze the direct and interactive relationships between safety climate and safety climate strength in relation to LOS, with exploratory analysis of other related outcomes among VLBW (< 1500 g) infants.

## Methods

This cross-sectional study links caregiver perceptions of safety climate to clinical outcomes data derived from a population-based clinical registry among 44 California NICUs.

### Sample and procedure

#### Selection of NICUs

A cross-sectional survey of safety climate and workforce engagement was offered to a voluntary sample of NICUs participating in a quality improvement initiative organized by the California Perinatal Quality Care Collaborative (CPQCC). (32) Of the 61 NICUs who participated in the improvement initiative, 44 participated in the survey, which was administered at the beginning of the improvement initiative (between June and September 2011).

Staff with a 0.5 full time equivalent or greater time commitment to the NICU for at least the four consecutive weeks prior to survey administration were eligible for inclusion. Paper-based surveys were administered during routine departmental and staff meetings. Respondents returned surveys to a locked box or sealable envelope to maintain confidentiality. Individuals not present in routine meetings were hand-delivered a survey, pencil, and return envelope. This administration technique has generated high response rates (13, 33) comparable to other studies of similar methodology. (34) CPQCC administered the survey and transmitted a de-identified data set to the authors for analysis.

#### Selection of patients

In order to capture outcomes concurrent with and subsequent to survey responses, clinical data routinely submitted to the CPQCC by Collaborative members reflecting VLBW infants born between January 1, 2011 and December 31, 2013 were linked to the survey data using unique identifiers for NICUs and patients. We used multiyear analysis due to the small number of VLBW infants cared for in some institutions.

### Measures

#### Survey data

For this study, we used the 7-item safety climate scale of the Safety Attitudes Questionnaire (SAQ) (13), scored for each individual on a 0–100 scale. For each NICU, the proportion of respondents achieving a score ≥ 75 out of 100 was calculated and reported as the percent positive response (PPR), in line with prior research. (35)

In addition, distribution of individual safety climate scores was plotted for each NICU, and summary statistics calculated, including mean, median, standard deviation (SD), and r_wg(j)_ (a measure of agreement, often used in inter-rater reliability assessments). The SD of individual safety climate scores within each NICU was used as the primary determination of its safety climate strength, in keeping with prior research. (24, 36, 37)

The survey also captured respondent characteristics including job position, years in specialty, gender, and predominant work shift. Job positions consisted of attending physicians, fellow physicians, neonatal nurse practitioners, registered nurses, and respiratory care practitioners.

#### Clinical data

CPQCC prospectively collects clinical data for infants born at 136 member hospitals using standard definitions developed by the Vermont Oxford Network, (38) and all data undergo a series of quality checks to ensure completeness and accuracy. Our primary outcome of interest was LOS. In line with other studies, (39, 40) we also evaluated post-menstrual age at discharge (PMA-DC) as a marker of LOS due to its closer approximation of a normal distribution and inherent adjustment for gestational age. LOS and PMA-DC were adjusted according to a prediction model developed in a previous study. (29) Covariates included sex, gestational age at birth, 5 min Apgar score (categorized as < 4, 4–6, or > 6), small for gestational age (<10th percentile), birth at the NICU under investigation (inborn) or outborn, birth weight, maternal race, and binary variables representing antenatal steroid use, fetal distress, major anomalies, and maternal hypertension.

Infants who died prior to discharge were excluded from the LOS analysis, but included for secondary outcomes. This is because early deaths result in short LOS, but cannot be considered a positive outcome. Furthermore, the LOS associated with death can vary widely with different clinical trajectories. Prior work has shown that mortality rates are not correlated with LOS among survivors, suggesting that this exclusion is unlikely to bias our results. (29)

We also calculated the secondary clinical outcomes of health care-associated infection (HAI), chronic lung disease (CLD), and mortality using standard CPQCC definitions. HAI includes any bacterial or fungal infection acquired after 3 days of age during the birth hospitalization. For infants transferred to another facility, attribution of infection was defined to include those acquired “here” and “here and elsewhere.” CLD is defined as oxygen requirement at 36 weeks post-menstrual age. We adjusted each secondary outcome according to a severity of illness model developed in a previous study. (35) Covariates included sex, gestational age at birth, 5 min Apgar score, small for gestational age (<10th percentile), and birth at the NICU of interest (inborn) or outborn.

### Data analyses

Descriptive statistics including frequencies, means, and SDs were used to describe survey responses and respondent demographics. Safety climate measures were calculated as described above, resulting in a safety climate PPR and safety climate strength for each NICU.

Following prior work on climate strength, (36, 37, 41) we explored the relationship between safety climate strength and safety climate level. Because safety climate strength and PPR may be mathematically related, as cautioned by Bliese and Halverson (42) and Schneider et al., (36) we calculated the Pearson’s correlation coefficient between the two measures, and plotted the relationships between strength and PPR for all NICUs. We evaluated the collinearity of strength and PPR as raw predictors and weighted predictors using variance inflation factors and the weighted correlation matrix, respectively.

To evaluate scale performance and to justify data aggregation to the NICU level of analysis, we calculated Cronbach’s alpha, the intraclass correlation coefficients ICC (1) and ICC (2), as well as the average r_wg(j)_ among all items. Cronbach’s alpha is a measure of scale reliability (acceptable range > 0.7). ICC (1) reflects the reliability of each individual provider’s assessment of their NICU’s mean safety climate (typical range 0.05–0.30), and ICC (2) reflects the reliability of unit means and thus the ability to distinguish among NICUs based on individuals’ responses (acceptable range > 0.7). (43) R_wg(j)_ is calculated at the NICU level and measures the degree to which individual responses within a NICU are consistent (acceptable range > 0.7). (44)

Basic descriptive statistics examined the variation in LOS and clinical outcomes across NICUs. We used infant-level ordinary least squares models and logistic regression models for associations of safety climate measures with risk-adjusted LOS and clinical outcomes. (35) Models also included covariates for California Children’s Services (CCS) level and birth year. Safety climate and safety climate strength terms were sequentially added to the models in the following order to illustrate any incremental effects of each factor: Safety climate PPR (Model 1), Safety climate PPR and safety climate strength (Model 2), and Safety climate PPR, safety climate strength, and the interaction between the two (Model 3). We used cluster-robust standard errors in all regressions, clustering by NICU. Analyses of LOS were performed using a log-normal marginal distribution due to its right-skewed distribution, and the Duan smearing retransformation (45) was used to obtain adjusted estimates from log-transformed regressions.

All statistical analyses were performed using SAS version 9.4. The study was approved by the Institutional Review Board at the first author’s university with waiver of informed consent.

## Results

### Descriptive characteristics

**Fig. 1:**
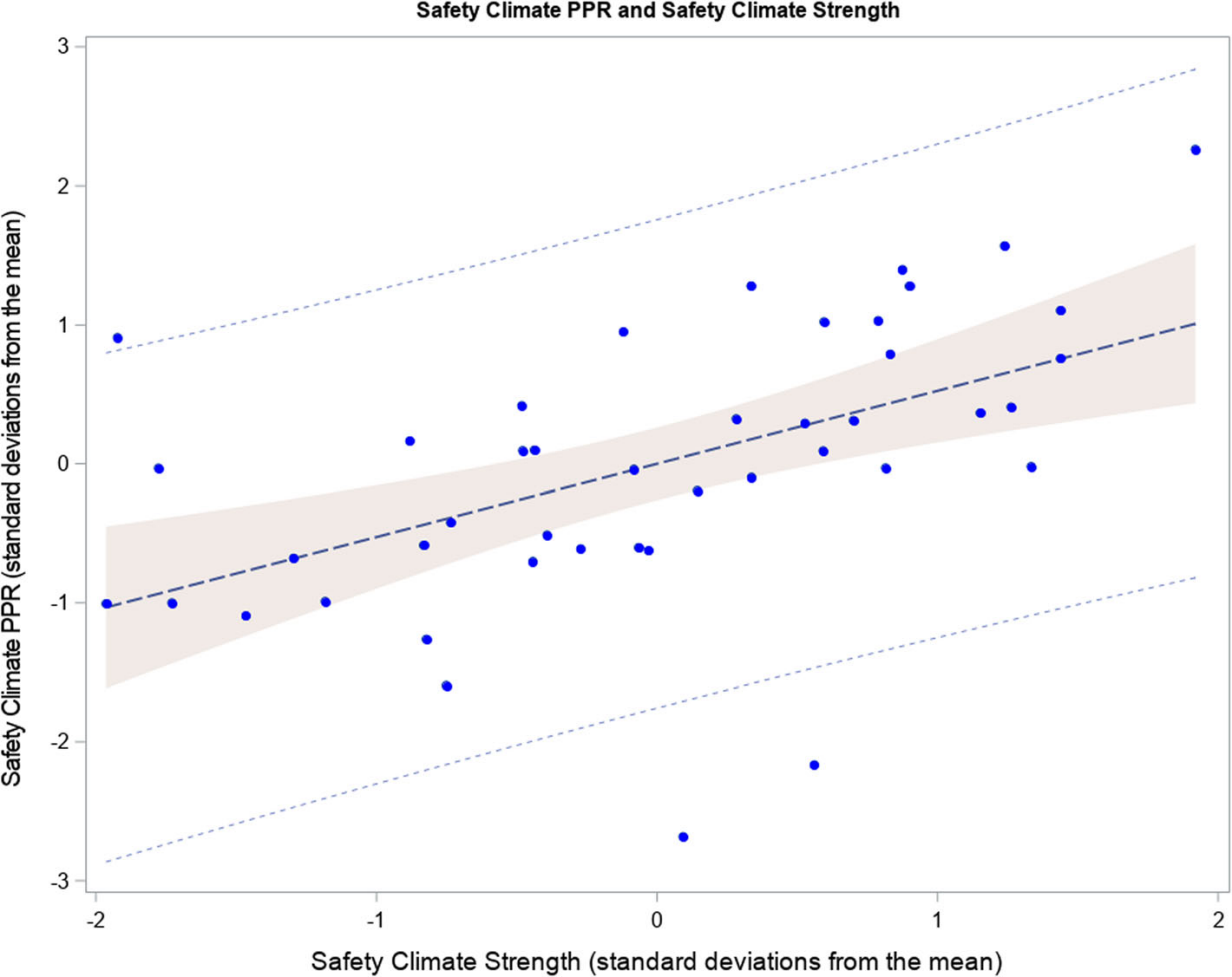
Relation between safety climate strength and safety climate percent positive response (PPR). *N* = 44 study NICUs. Safety climate strength calculated as intra-unit standard deviation (SD) of safety climate scores with higher values indicating higher safety climate strength. PPR and SD presented as standard deviations from the mean PPR: percent positive response.

Forty-four NICUs participated in this study, with 2073 of 3294 surveys returned for a 62.9% response rate. Individual NICU response rates averaged 69.7% (SD 19.8, range 22–100%), safety climate PPRs ranged from 33 to 95% (mean 65.9 ± 11.7, median 66.3, IQR 58.2–72.2), and safety climate strength ranged from 10.6 to 22.8 (mean 16.6 ± 3.1). Safety climate strength and PPR were positively correlated, as shown in Fig. 1 (*r* = 0.61, *P* < .001), but with acceptable variance inflation factors of 1.57 and 1.58, respectively, in multivariable modeling.

Table 1 lists respondent characteristics, which indicated 60% of all respondents with 11 or more years in their specialty, and 2.4% of all respondents with less than 1 year of experience. Across all respondents, the mean safety climate score was 76.5 ± 17.5, with 63.6% of respondents meeting the threshold score of 75/100. The safety climate score distributions of representative NICUs with stronger and weaker climates are shown in Additional file 1: Figure S1.

**Table 1:** Description of survey respondents and clinical sample

	N (%) or mean (±SD)
NICUs, *N*	44
Level of care	
Intermediate	6 (14)
Community	27 (61)
Regional	11 (25)
Survey response rate	70% (±20)
Safety Climate PPR	66% (±12)
Safety Climate strength	17 (±3.1)
Respondents, *N*	2073
Females	1697 (85)
Typical shift	
Days	894 (48)
Nights/Evenings	681 (36)
Variable	293 (16)
Position	
Physician	235 (12)
Neonatal nurse practitioner	35 (1.7)
Registered nurse	1464 (72)
Respiratory therapist	286 (14)
Other	21 (1.0)
Years in specialty	
< 1	47 (2.4)
1–4	266 (13)
5–10	476 (24)
11–20	538 (27)
> = 21	643 (33)
VLBW infants, *N*	7338
Gestational age, weeks	28.2 (±2.9)
Birthweight, grams	1061 (±285)
Small for gestational age	1392 (19)
Male sex	3701 (50)
5-min APGAR score	
< 4	449 (6.1)
4–6	1298 (18)
> 6	5591 (76)
Inborn	5686 (77)
Length of stay (all infants), days	66 (±40)
Length of stay (survivors), days	68 (±37)
PMA-DC (all infants), weeks	38 (±5.0)
PMA-DC (survivors), weeks	39 (±3.8)
Mortality	653 (8.9)
Chronic lung disease	1415 (25)
Health care-associated infection	640 (8.7)

### Scale performance and data aggregation

Cronbach’s alpha for the 7-item safety climate scale was 0.81. ICC (1) was 0.06 and ICC (2) was 0.75, suggesting that data aggregation at the level of the NICU is appropriate. (43) Similarly, the average rwg(j) of the safety climate scale across NICUs was 0.86 (with a range of 0.68 to 0.93) further suggesting the appropriateness of aggregation to the NICU level.

### Direct and interactive relationships between safety climate and safety climate strength in relation to LOS

Table 1 shows the characteristics of the clinical sample. Of the 7338 VLBW infants included in the study, 653 (8.9%) died prior to discharge and 3 had incomplete LOS information, resulting in 6682 infants for the primary analysis. Mean LOS was 68 days, with a median LOS of 60 days (IQR 41 to 88 days).

**Fig. 2:**
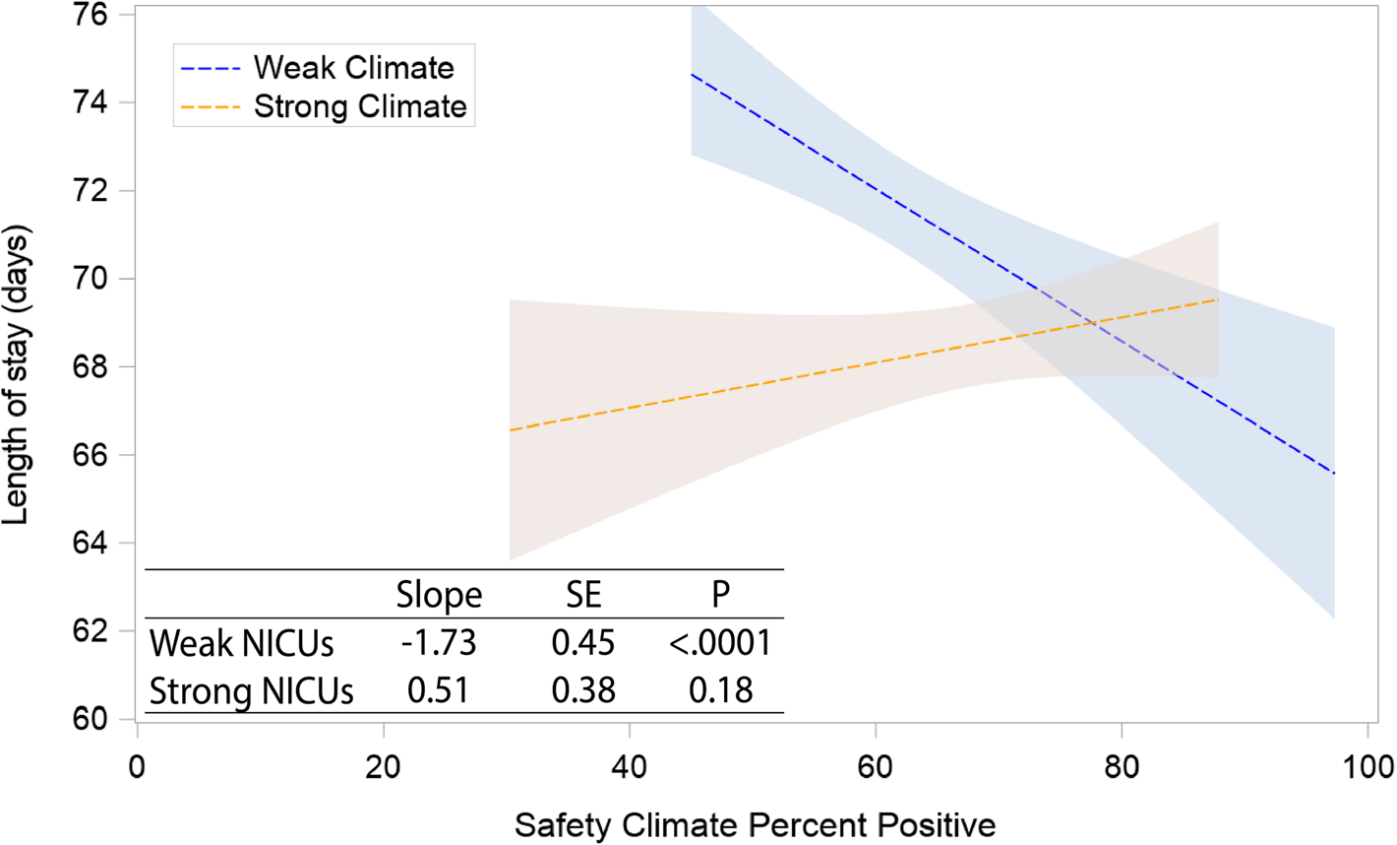
Safety climate strength and the relation between percent positive response and length of stay. Effect of safety climate strength on the relation between safety climate percent positive response (PPR) and risk-adjusted length of stay among very low birthweight infants

Table 2 shows patient-level associations with LOS after adjustment for clinical characteristics. All three models are shown, with sequentially increasing complexity including evaluation of safety climate PPR (model 1), safety climate PPR and safety climate strength (model 2), and their interaction (model 3). Safety climate PPR was not associated with LOS in models 1 or 2, but safety climate strength was associated with LOS in models 2 and 3. Addition of safety climate strength as an interaction term revealed divergent associations as illustrated in Fig. 2, modeled at ±1 SD from the mean climate strength. NICUs with weak climate (1 SD below the mean climate strength) and low PPR had LOS 3 days higher than the mean, and each 10% increase in PPR associated with a 1.73-day decrease in LOS. NICUs with strong climate (1 SD above the mean climate strength) and low PPR had LOS 1 day lower than the mean, and each 10% increase in PPR associated with a non-significant trend toward increase in LOS. Weak NICUs with low PPR had longer LOS than strong NICUs with low PPR (*P* < .001), but there was no difference between weak and strong NICUs with high PPR (*P* = .07).

**Table 2:** Relationship between safety climate, safety climate strength and length of stay

	Parameter estimate	SE	Incremental F	*P*	RMSE
Model 1 (CF + PPR)					
Safety Climate PPR	− 0.38	0.31	1.52	.22	23.23
Model 2 (CF + PPR + Strength)					
Safety Climate PPR	0.62	0.39	2.58	.11	23.20
Safety Climate Strength	−1.59	0.37	18.59	<.001	
Model 3 (CF + PPR + Strength + PPR*Strength)					
Safety Climate PPR	5.20	1.79	8.48	.004	23.19
Safety Climate Strength	−5.92	1.69	12.27	<.001	
Safety Climate PPR * Strength	−5.58	2.12	6.89	.009	

*n* = 6682 infants in 44 NICUs. Ordinary least squares regression analysis at the patient level, with LOS transformed to log-normal scale.

*RMSE* root mean square error, reflecting standard deviation of the unexplained variance. Lower values indicate better model fit.

*LOS* Length of stay, *PPR* Percent positive response.

*CF* Clinical factors: sex, gestational age, 5 min Apgar score, small for gestational age, outborn, birth weight, antenatal steroids, fetal distress, major anomalies, maternal hypertension, and maternal race.

#### Sensitivity analyses

The results did not differ materially in sensitivity analyses, shown in Additional file 1: Table S1. These analyses used r_wg(j)_ as the indicator of safety climate strength, used PMA-DC as the marker for LOS, and included patient deaths in the LOS analysis, respectively. Stratification of patient outcomes by birth year is shown in Additional file 1: Figure S2.

#### Secondary outcome measures

**Fig. 3:**
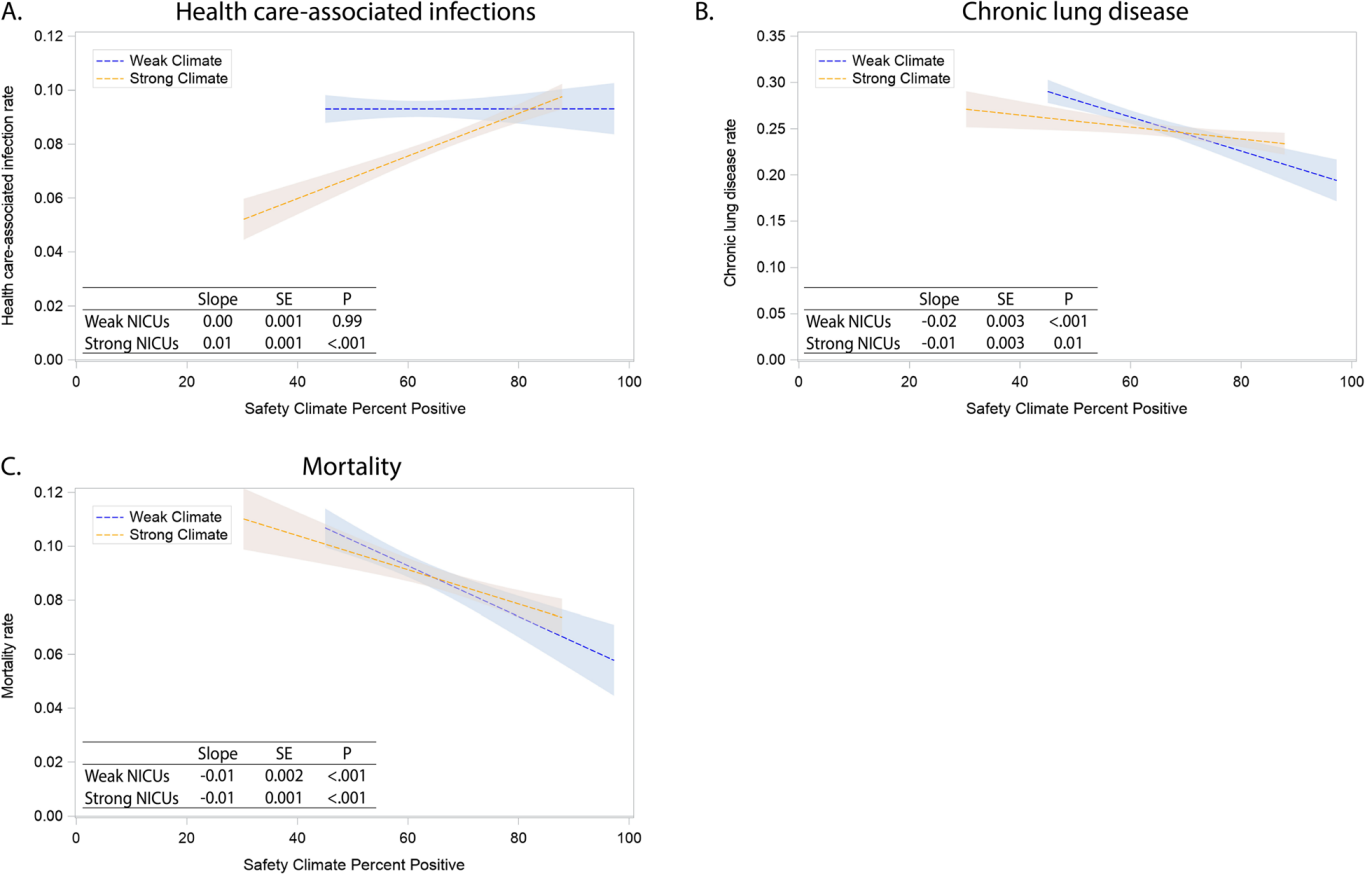
Safety climate strength and the relation between percent positive response and secondary outcomes. Effect of safety climate strength on the relation between safety climate percent positive response (PPR) and risk-adjusted **a**. health care-associated infections, **b**. chronic lung disease, and **c**. mortality

Figure 3 and Additional file 1: Table S2 show the associations of safety climate PPR and safety climate strength with other clinical outcomes. In a similar pattern to the primary analysis, NICUs with strong climates and low PPR exhibited the lowest odds of risk-adjusted HAI. CLD exhibited no direct effects for PPR or climate strength in the absence of the interaction, but a marginally significant interaction effect. PPR and climate strength were significantly related to mortality directly and in interaction modeling. For both CLD and mortality, higher PPR associated with improved outcomes in interaction modeling, but the climate strength interaction did not translate to clinically meaningful differences.

## Discussion

This study found that safety climate PPR and safety climate strength are associated with LOS among VLBW infants. Specifically, safety climate strength moderates the association between safety climate PPR and LOS among VLBW infants in divergent ways.

Historically, safety climate has been considered a score on a 100-point scale assuming a shared consensus of local emphasis on patient safety. In this *direct consensus model*, variation in responses is considered a nuisance of imprecise measurement. However, a *dispersion model* views the variability of responses as a focal construct. (36) Ginsburg and Oore recently recommended a multifaceted approach to safety climate analysis, including safety climate level, safety climate strength, and histogram analysis. (24) Our analysis attempts to apply this approach by combining safety climate level (measured as PPR) and safety climate strength (measured as SD) to explain variation in NICU LOS.

Although safety climate PPR and safety climate strength showed moderate association with each other, safety climate strength moderated the association between PPR and LOS in divergent ways. While the interpretation of safety climate PPR is straightforward, the interpretation of safety climate strength merits further consideration. It is possible that two distinct principles affect safety climate strength: (1) the average level of safety climate perceptions within the unit and (2) the true consistency of safety culture underlying the safety climate perceptions.

Due to the ceiling effects with a limited response scale, NICUs with high safety climate PPR are expected to also exhibit agreement among respondents. For instance, upper limits were much more frequently encountered than the lower limits (7.6% of respondents scored 100/100, while no respondents scored 0/100). Consistent with this, high safety climate PPR scores corresponded with less variability among respondents (i.e., stronger safety climate). Conversely, NICUs with lower safety climate PPR showed greater variability in general (i.e., weaker safety climate), as even NICUs with poor aggregate safety climate had some individuals with positive safety climate perceptions. As an illustration, at least one-third of respondents reported positive safety climate in all NICUs, and every NICU had at least one respondent scoring > 95/100.

However, NICUs exhibited differences in safety climate strength not fully explained by the safety climate PPR, suggesting that the *strength* of a safety climate does not only correspond to its *level*. Consistency and convergence of perceptions about safety climate, i.e., safety climate strength, can result from objective and social factors. The shared conditions of the workplace (e.g., actual safety performance) and interdependent work can homogenize perceptions. The process through which this occurs is referred to as social information processing, whereby ongoing social influence and learning occurs through help-seeking and other work-related interactions. (46) Convergent attitudes can cement over time through attraction-selection-attrition processes that increase similarity in the priority placed on safety by favoring selection and retention of employees who value a positive safety climate. (47) For example, a NICU may have inadequate resources to promote safety, and workers may discuss this lack of resources as they carry out their interdependent work. In this scenario, the social information processing at work in the NICU results in both low safety climate PPR and high consensus about the low safety climate, increasing safety climate strength. It is the independent and joint consequences of safety climate PPR and safety climate strength that have been evaluated in this study.

The observed association between safety climate strength and LOS suggests that safety climate not only affects typical safety domains, but also efficiency of care. When appropriately risk-adjusted, LOS serves as a straightforward indicator of composite outcomes. A wide range of adverse events are expected to increase LOS, including medication errors, surgical errors, and HAIs. However, LOS serves as a marker of efficiency beyond mere prevention of errors. In this way, our findings suggest that a strong safety climate may be indicative of strong shared basis for action that facilitates coordination and teamwork among NICU providers, resulting in more safely and swiftly transitioning infants from dependency to discharge. Furthermore, the magnitude of this association is practically significant, with average LOS differences of several days in relation to safety climate strength. For the median-sized NICU in this study, a 1-day reduction in average LOS among VLBW infants translates to 119 fewer patient-days per year, representing hundreds of thousands of health care dollars saved and better resource availability for infants with higher need. (28) Among neonatology quality improvement, reducing LOS of VLBW infants by 1–5 days is considered a significant improvement, particularly considering that LOS is partially dictated by physiological maturity around 34–35 weeks post-menstrual age, limiting the degree of reduction that could be expected. (48–51)

In addition, we find an interaction effect between safety climate PPR and safety climate strength, suggesting the value of considering safety climate strength. Specifically, and unexpectedly, we find NICUs with strong safety climate but low safety climate PPR exhibited the earliest discharges and lowest rates of HAIs. Although contrary to expectations, we speculate that this surprising relationship results from a motivation to improve that may result from the shared perception that we are not safe around here and we all agree on that (i.e., the combination of perceiving low safety climate PPR and strong safety climate). That particular combination means that at least the preponderance of employees perceive a shared reality in which the safety climate is in need of improvement. The NICUs in the present study were participants in a voluntary quality improvement project, and thus may have been especially likely to connect low safety climate PPR with a need for improvement. The corresponding sense of urgency that often accompanies shared perceptions of problematic conditions fosters collective, coordinated behavioral change that can improve outcomes. (52) This sense of urgency may not be present in NICUs with high PPR, even if exhibiting a strong climate. In contrast, low safety climate PPR with weak safety climate means employees may be experiencing the climate in divergent ways and find it difficult to generate appropriate, coordinated responses. Lack of coordination can slow care delivery, increase likelihood of adverse events, and extend LOS.

Although evaluation of a causal link between safety climate strength and quality of care is in its infancy, the characteristics that have been identified as enhancing climate strength are also likely to foster behavior that expedites high quality care delivery and reduces LOS including unit cohesion, stability, and dense communication networks. (53–55) Leader actions also contribute to safety climate strength. Specifically, transformational leadership grounded in higher quality exchanges between leaders and subordinates can generate stronger safety climates and better outcomes through individualized interaction, (53, 56, 57) providing more information, (58, 59) and making priorities clear by offering feedback and recognition for safety-related behaviors. (60) Leaders can also cultivate their ability to enhance safety climate PPR and strength by improving their safety-related interactions through data, feedback, and tools. (59, 61, 62) One such example is Leadership WalkRounds with feedback, which has been shown to have profound impacts on safety climate and provider engagement. (63, 64) In summary, when unit members interact more and when leaders communicate more fully to state and reinforce safety priorities, climate strength increases. (26)

It should be noted that although the details varied for the secondary outcomes, safety climate strength still contributed important detail to the interpretation of PPR alone. Individually, these are relatively infrequent outcomes among VLBW infants, and this study was not adequately powered to detect subtle differences in these outcomes. Thus, it remains plausible that safety climate PPR and safety climate strength are associated with these outcomes, but larger scale studies are needed to evaluate this hypothesis.

This study must be interpreted in the context of its design. As a cross-sectional study, our research cannot determine causality of the observed associations and suggests the need for future longitudinal research that measures changes in safety climate over time. Participation in this study was limited and available on a first-come basis, raising the possibility of response bias at the NICU level. However, such a bias would make it more likely that participating NICUs had high safety climate PPR (and relatively stronger climate), making it less likely to find significant effects. Selection bias among respondents is also possible and we were unable to compare respondent demographics to non-respondent demographics, although our response rate of 63% compares favorably with acceptable thresholds for response rates and other studies of safety climate, including studies validating the Safety Attitudes Questionnaire. (13, 34, 65, 66) The study sample also mitigates the concerns of bias as it offers a large, diverse, and representative sample of NICUs across the state of California. As such, the findings are more readily able to generalize to NICUs. Although we have used extensive risk-adjustment in line with prior research, (29, 67) LOS is highly dependent on baseline clinical characteristics and it is possible unmeasured confounders remain. As a hypothesis-generating study, no correction was made for multiple testing. Because our phenomena of interest (safety climate PPR and safety climate strength) occur at the NICU level, we did not use a random effects approach to account generally for unit-level variation, potentially excluding relevant NICU-level effects unrelated to safety climate. However, we did include a wide set of unit and patient-level controls, and employed cluster-robust standard errors to mitigate this risk.

## Conclusion

Prior research in health care has emphasized safety climate PPR largely to the exclusion of the consistency of perceptions of safety climate, i.e., safety climate strength. We find that omission is costly, as safety climate strength has both a direct and moderating effect on LOS among VLBW infants. The specific form of the interaction further suggests that for units with low safety climate PPR and low safety climate strength, they might benefit most by building awareness and cultivating consistent assessments as a basis for engagement in patient safety interventions. Evaluations of NICU safety climate should thus diagnose and account for the distribution across respondents. Our findings also demonstrate that promoting safety climates with high PPR and strength within NICUs provides benefits beyond safety to the efficiency of care for these vulnerable patients.

## Supplementary information

**Additional file 1: Figure S1.** Representative safety climate score distributions for NICUs with stronger and weaker safety climate strengths. **Table S1.** Relationship between safety climate, safety climate strength, and length of stay. **Table S2.** Relationship between safety climate and secondary clinical outcomes. **Figure S2.** Effect of safety climate strength on the relation between safety climate percent positive response (PPR) and risk-adjusted length of stay among very low birthweight infants, stratified by birth year.

## Data Availability

The data that support the findings of this study are available from the California Perinatal Quality Care Collaborative, but restrictions apply to the availability of these data, which were used under license for the current study, and so are not publicly available. Data are however available from the first author (dtawfik@stanford.edu) upon reasonable request and with permission of the California Perinatal Quality Care Collaborative.

## Abbreviations

CLD: Chronic lung disease
CPQCC: California Perinatal Quality Care Collaborative
HAI: Health care-associated infection
LOS: Length of stay
MD: Physician
NICU: Neonatal intensive care unit
NNPs: Neonatal nurse practitioners
PMA-DC: Post-menstrual age at discharge
PPR: Percent positive response
RN: Registered nurse
SAQ: Safety Attitudes Questionnaire
VLBW: Very low birth weight
