# Supplementary material for: Safety climate, safety climate strength, and length of stay in the NICU
Source: BMC Health Serv Res. 2019 Oct 22;19:738. doi: 10.1186/s12913-019-4592-1 (PMC6805564; doi:10.1186/s12913-019-4592-1)
Supplement: Supplementary file 1 — Additional file 1: Figure S1. Representative safety climate score distributions for NICUs with stronger and weaker safety climate strengths. Table S1. Relationship between safety climate, safety climate strength, and length of stay. Table S2. Relationship between safety climate and secondary clinical outcomes. Figure S2. Effect of safety climate strength on the relation between safety climate percent positive response (PPR) and risk-adjusted length of stay among very low birthweight infants, stratified by birth year. [file 12913_2019_4592_MOESM1_ESM.docx]

**Supplemental Figure 1.** Representative safety climate score distributions for NICUs with stronger and weaker safety climate strengths.


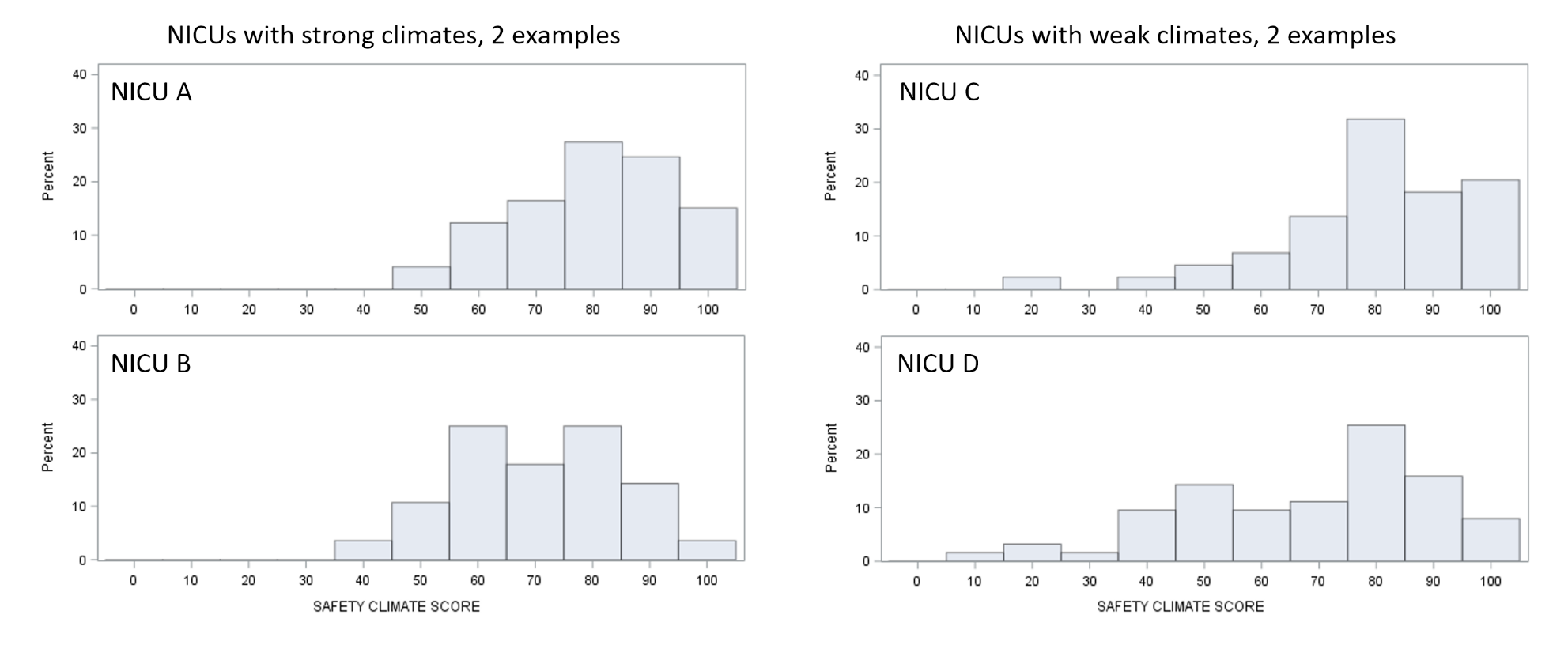


**Supplemental Table 1.** Relationship between safety climate, safety climate strength, and length of stay. Sensitivity analyses using r_wg(j)_ as marker of safety climate strength, post-menstrual age at discharge as marker of length of stay, and patient deaths included in length of stay analysis.

| **Dependent variable** | **Independent variable** | **Incremental F** | ***P*** |
| --- | --- | --- | --- |
| Length of stay | Safety Climate PPR | 1.04 | .31 |
|  | Safety Climate r_wg(j)_ | 7.45 | .006 |
|  | Safety Climate PPR * r_wg(j)_ | 4.06 | .04 |
| Post-menstrual age at discharge | Safety Climate PPR | 6.76 | .009 |
|  | Safety Climate Strength | 9.69 | .002 |
|  | Safety Climate PPR * Strength | 4.54 | .03 |
| Length of stay (including patient deaths) | Safety Climate PPR | 0.93 | .33 |
|  | Safety Climate Strength | 3.51 | .06 |
|  | Safety Climate PPR * Strength | 2.59 | .11 |
| n = 6682 infants in 44 NICUs (7335 infants in model including patient deaths). Ordinary least squares regression analysis at the patient level, with length of stay and post-menstrual age at discharge transformed to log-normal scale.  PPR – Percent positive response  All models adjusted for clinical factors: sex, gestational age, 5 minute Apgar score, small for gestational age, outborn, birth weight, antenatal steroids, fetal distress, major anomalies, maternal hypertension, and maternal race. | | | |

**Supplemental Table 2.** Relationship between safety climate and secondary clinical outcomes.

|  | Incremental F | *P* | Hosmer-Lemeshow goodness-of-fit |  |
| --- | --- | --- | --- | --- |
| Health care-associated infections |  |  |  |  |
| Safety Climate PPR | 7.85 | .005 | χ^2^ = 19.84  DF = 8  *P* value = .01 |  |
| Safety Climate Strength | 7.20 | .007 |  |  |
| Safety Climate PPR * Strength | 5.36 | .02 |  |  |
| Chronic lung disease |  |  |  |  |
| Safety Climate PPR | 2.50 | .11 | χ^2^ = 2.45  DF = 8  *P* value = .96 |  |
| Safety Climate Strength | 3.55 | .06 |  |  |
| Safety Climate PPR * Strength | 4.12 | .04 |  |  |
| Mortality |  |  |  |  |
| Safety Climate PPR | 6.34 | .01 | χ^2^ = 12.37  DF = 8  *P* value = .14 |  |
| Safety Climate Strength | 7.86 | .005 |  |  |
| Safety Climate PPR * Strength | 8.31 | .004 |  |  |
| n = 7338 infants in 44 NICUs. Multivariable logistic regression, analysis at the patient level.  PPR – Percent positive response  DF – degrees of freedom  All models adjusted for clinical factors (sex, gestational age, 5 minute Apgar score, small for gestational age, outborn) and perceptions of management, California Children’s Services (CCS) level, number of VLBW infants, birth year, survey response rate, and number of survey respondents. | | | | |

**Supplemental Figure 2.** Effect of safety climate strength on the relation between safety climate percent positive response (PPR) and risk-adjusted length of stay among very low birthweight infants, stratified by birth year.
